# New Gene Signature–Based Prognostic Model for Patients With VEGF‐Overexpressing Esophageal Squamous Cell Carcinoma

**DOI:** 10.1155/bmri/5694628

**Published:** 2025-12-07

**Authors:** Yonghui Li, Ruiyao Wang, Haibo Wang, Tingting Li, Cheng Long Zhang, Shaoyong Dong, Biao Zhang

**Affiliations:** ^1^ Department of Thoracic Surgery, Affiliated Hospital of Hebei University, Baoding, Hebei, China, hbu.cn

**Keywords:** CAFs, ESCC, prognosis model, TCGA, VEGF

## Abstract

**Background and Aims:**

The effects of vascular endothelial growth factor (VEGF) overexpression on the prognosis of patients with esophageal squamous cell carcinoma (ESCC) are still unclear. The aim of this study was to construct and evaluate a mutant gene–based model to predict the prognosis of ESCC patients with VEGF overexpression.

**Methods:**

Samples from 50 ESCC patients with VEGF overexpression were subjected to next‐generation sequencing (NGS) to identify gene mutations. The associations between the enrichment of these mutations and patient outcomes were also evaluated in a cohort from The Cancer Genome Atlas. Hazard ratios were identified via the Kaplan–Meier and Cox analyses. A support vector machine recursive feature elimination algorithm was used to construct the model, and receiver operating characteristic analysis was carried out to evaluate its prognostic performance.

**Results:**

ESCC patients with *FAT1*, *FGF3*, *FGF12*, and *FGF19* mutations; advanced M stage; and high neutrophil counts tended to have poorer prognoses.

**Conclusion:**

A model based on a four‐gene signature effectively predicts the prognosis of ESCC patients.

## 1. Introduction

Esophageal squamous cell carcinoma (ESCC) is among the most prevalent malignant carcinomas worldwide [1], with epidemiologic studies indicating that approximately 79% of all ESCC cases occur in Asia [2]. Despite the availability of multiple chemotherapy regimens, including neoadjuvant and preoperative therapies, the 5‐year survival rate among ESCC patients is less than 10% and rarely exceeds 40%, and the median overall survival (OS) is less than 1 year [3]. Clinical studies have revealed individual differences in patient responses to therapeutic strategies [4, 5], as well as marked regional variations in the genetic landscape of ESCC. These findings suggest a need for further exploration to guide the development of more individualized treatments [6].

VEGFs are a large family of polypeptides with central roles in many cell signaling pathways [7]. Studies have reported conflicting findings regarding the role of VEGF in the clinicopathology of ESCC patients [8–10], possibly because different genetic variants combine with abnormal expression of VEGF to exert particular effects on different tumor cells [11]. Thus, it is necessary to elucidate the specific cellular functions of the most common genetic polymorphisms against a background of abnormal VEGF expression in ESCC.

Owing to the heterogeneity of ESCC, the mechanisms underlying the effects of mutations may be highly complex. Thus, gene signatures may be particularly suitable for use in the prediction of patient prognosis in ESCC, as they can specifically reflect multiple genetic changes and their associations with outcomes [12]. To date, more than 30 different mutational signatures have been recorded in the Catalogue of Somatic Mutations in Cancer and have been shown to predict various outcomes. For example, objective response and progression‐free survival (PFS) rates were found to be higher in ESCC patients with *CCND1*, *FGF3*, *FGF4*, and *FGF19* amplification than in those without [13], and a recent study proposed a combined prognostic model for ESCC progression and metastasis consisting of *KMT2D* mutation; *CCND1*, *FGF3*, *FGF4*, and *FGF19* amplification; and *CDKN2A* deletion [14]. To identify such signatures, it is important to identify hub genes that are associated with the oncogenesis and development of ESCC across various datasets. Thus, in the present study, we developed a prognostic gene signature for ESCC patients with VEGF overexpression via machine learning–based analysis of next‐generation sequencing (NGS) results, data from The Cancer Genome Atlas (TCGA), and clinical datasets. We also evaluated the prognostic value of the gene signature in ESCC patients.

## 2. Materials and Methods

### 2.1. Patients, Treatment, and Follow‐Up

This study used clinical data and stored tumor tissues from 50 ESCC patients who were admitted to the Affiliated Hospital of Hebei University between April 2017 and December 2022. All enrolled participants had been diagnosed with stage I–III cancer according to the seventh edition of the American Joint Committee On Cancer (AJCC) staging manual. The hospital’s ethics board approved the study (Approval No. 20220803). The inclusion criteria for patients were as follows: (1) newly diagnosed with ESCC and > 18 years of age; (2) able to complete treatment and 5‐year follow‐up; (3) well‐documented treatment, recurrence, recurrence‐free survival (RFS), and OS information available; (4) Eastern Cooperative Oncology Group (ECOG) score of 0–1; (5) absolute neutrophil count ≥ 1.5 × 10^9^/L, platelet count ≥ 90 × 10^9^/L, hemoglobin content ≥ 9.0 g/dL, blood protein level ≥ 2.8 g/L, total bilirubin ≤ 1.5 × upper limit of normal (ULN), ALT and AST values ≤ 2.5 × ULN, serum creatinine ≤ 1.5 × ULN, and international normalized ratio < 1.5 × ULN; and (6) detectable VEGF expression in tumors. The exclusion criteria were as follows: (1) had recurrent adenocarcinoma before admission, (2) had received therapies before admission, or (3) had any other observed clinical disorders. RFS is a more sensitive measure than OS in terms of patient response to therapy. Therefore, we used RFS as an indicator of the prognosis of participants after treatment. All enrolled patients were informed of the experimental protocol and signed informed consent forms. Participants who died from causes unrelated to ESCC were excluded from the survival analysis.

### 2.2. Analysis of TCGA Data

Data were obtained from TCGA for 132 ESCC patients whose VEGF expression was greater than the mean value who had received drug treatment. These data, which included information on VEGF mutations and expression, as well as patient clinical characteristics, were extracted and downloaded via a dedicated web server. All 132 patients had records of having been diagnosed with clinical stage I–III disease. No RFS data for these patients were available via the TCGA; therefore, we used PFS data to evaluate the prognostic value of the gene signature.

### 2.3. NGS

DNA (100 ng) was isolated from tumor tissues and used to construct an NGS library. Capture‐based targeted sequencing was performed using a panel of 520 cancer‐related genes (OncoScreen plus, Burning Rock Biotech, Guangzhou, China). The DNA concentration was quantified via a Qubit 2.0 fluorometer (Thermo Fisher Scientific, Waltham, Massachusetts, United States), and the DNA quality was determined by measuring the 260/280 and 230/260 absorbance ratios via a spectrophotometer (Nanodrop ND‐1000, Peqlab Biotechnologie, Erlangen, Germany). The loading concentration of the library was then adjusted to 10 ng/*μ*L, and the library was sequenced via a NextSeq 550 AR sequencer (Illumina, Inc. San Diego, CA, United States) with paired‐end reads. The average sequencing depth was 1000× per sample. All the sequencing data have been uploaded to the Figshare repository (10.6084/m9.figshare.24793695.v1/). Single‐nucleotide polymorphisms (SNPs) and other variations were called according to the hg38 (GRCh38) version of the human genome.

### 2.4. Selection of Genetic Polymorphisms

Mutant site calling was performed with GATK v.3.2 and VarScan v.2.4.3, and the SNPinfo web server (http://snpinfo.niehs.nih.gov/) was used to identify functional SNPs in the sequences. The selection criteria were a minor allele frequency ≥ 5*%* and a linkage disequilibrium coefficient (*r* ^2^) < 0.8. All the SNPs were associated with ESCC. The tumor mutation burden was analyzed via the R maftools package. The gene set shared between the TCGA and NGS sequence data was determined via a Venn diagram.

### 2.5. Gene Set Enrichment Analysis (GSEA)

To identify cell signaling pathways that are potentially associated with the gene signature, we used GSEA to assess the enrichment of Kyoto Encyclopedia of Genes and Genomes (KEGG) pathways. The two datasets (TCGA and NGS) were divided into high‐ and low‐expression subgroups according to the expression levels of selected hub genes. The Top 10 significantly enriched terms for each dataset were identified via the following threshold values: false discovery rate < 0.25, *p* < 0.05, and |normalized enrichment score| > 1).

### 2.6. Identification of the Optimal Prognostic Gene Signature via the Support Vector Machine Recursive Feature Elimination (SVM‐RFE) Model

The expression data for the selected genes and patient clinical characteristics were merged. The Shapiro–Wilk test revealed that gene expression among the study participants followed a normal distribution. Using RFS as the outcome, we established an SVM‐RFE model with 10‐fold cross‐validation via an SVM package to screen variables (genes) with respect to their prognostic value. We then constructed a logistic regression model to predict the RFS of ESCC patients on the basis of the resulting four‐gene signature via the prediction function in the R glm package. The pROC package was used for receiver operating characteristic (ROC) curve visualization and statistical validation. All analyses were performed in R v. 4.3.1.

### 2.7. Statistics

The RFS and PFS rates of the two study cohorts were compared via the log‐rank test, and Kaplan–Meier curves were plotted to assess the cumulative probability of survival in ESCC patients. One‐way analysis of variance combined with Tukey′s test was used for comparisons among multiple groups. Spearman′s rank correlation was used to assess associations of signature gene expression with detected genomic alterations and clinicopathological characteristics. A two‐sided *p* value < 0.05 was considered to indicate statistical significance.

## 3. Results

### 3.1. NGS Results

**Figure 1:**
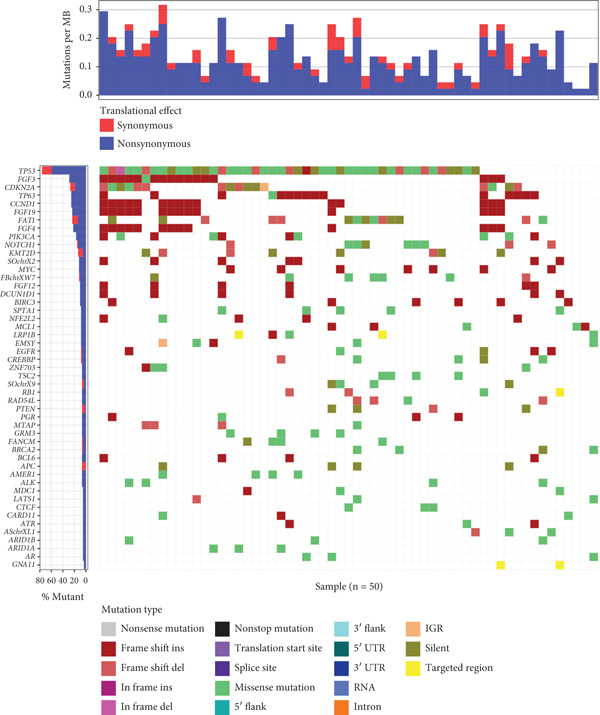
Gene mutations associated with VEGF expression in the enrolled clinical patient population. (a) Waterfall plot of the Top 10 mutated genes in the ESCC cohort with VEGF expression.

Cancer and paracancerous tissues were collected during surgery from ESCC patients with VEGF overexpression, and a targeted 520‐oncogene NGS panel was used to identify gene mutations in these tissues. The Top 10 most frequently mutated genes were *TP53*, *FGF3*, *CDKN2A*, *TP63*, *FGF19*, *FAT1*, *FGF4*, *PIK3CA*, *FGF12*, and *NOTCH1* (Figure 1). Cox analysis was subsequently used to evaluate the associations of patient clinical characteristics and mutations in these genes with RFS rates. According to the multivariate analysis, AJCC stage III/IV disease and mutations in *FGF3*, *TP63*, *FGF19*, and *FAT1* were associated with significantly increased hazard ratios among patients with ESCC (Table 1,  ^∗^ *p* < 0.05). Notably, four of these genes (*FGF3*, *FGF19*, *FGF12*, and *FGF4*) are members of the fibroblast growth factor (FGF) family.

**Table 1:** Cox regression analysis of many clinical–pathological characteristics and Top 10 genetic mutations in *VEGF* expression ESCC subjects.

**Variable**	**HR**	**Cl (95%)**	**p**
Multivariate analysis (*n* = 50)			
Age	1.790	1.325–3.652	0.087
Gender	0.582	0.157–2.127	0.149
T stage (T1–2/T3–4)	2.311	1.985–3.767	0.086
N stage (N0/N1–X)	2.011	1.668–3.121	0.044
M stage (M0/MX)	1.122	0.385–2.117	0.054
Clinical stage (III–V/I–II)	1.889	1.112–4.886	0.013 ^∗^
TP53 (with/without)	1.071	0.668–3.515	0.117
FGF3 mutation (with/without)	2.031	1.232–2.879	0.013 ^∗^
CDKN2A mutation (with/without)	1.096	0.554–0.371	0.442
TP63 mutation (with/without)	1.886	0.023–1.115	0.021 ^∗^
CCND1 mutation (with/without)	2.001	0.011–4.882	0.231
FGF9 mutation (with/without)	1.129	0.117–3.007	0.031 ^∗^
FAT1 mutation (with/without)	1.889	0.557–2.665	0.008 ^∗^
FGF4 mutation (with/without)	1.353	0.251–2.776	0.068
PI3KCA mutation (with/without)	1.085	0.772–3.662	0.117
Notch1 mutation (with/without)	1.971	0.782–2.525	0.666
WHO histological classification	1.175	0.515–2.797	0.525
Univariate analysis (*n* = 50)			
Age	0.889	0.257–1.131	0.112
Gender	1.212	0.767–2.022	0.093
T stage (T1–2/T3–4)	1.078	0.223–1.667	0.068
N stage (N0/N1–X)	1.913	0.887–3.252	0.038 ^∗^
M stage (M0/MX)	1.787	1.005–2.668	0.047 ^∗^
Clinical stage (III–IV/I–II)	2.013	1.746–3.889	0.014 ^∗^
TP53 (with/without)	2.218	1.996–3.223	0.055
FGF3 mutation (with/without)	1.848	0.973–2.994	0.022 ^∗^
CDKN2A mutation (with/without)	1.212	0.342–1.559	0.387
TP63 mutation (with/without)	1.747	0.212–2.935	0.019 ^∗^
CCND1 mutation (with/without)	2.121	0.485–4.082	0.197
FGF9 mutation (with/without)	1.224	0.687–2.697	0.029 ^∗^
FAT1 mutation (with/without)	1.889	0.666–1.897	0.028 ^∗^
FGF4 mutation (with/without)	1.552	0.483–2.503	0.041 ^∗^
PI3KCA mutation (with/without)	0.989	0.655–2.993	0.088
Notch1 mutation (with/without)	1.783	1.022–2.799	0.035 ^∗^
WHO histological classification	0.882	0.479–2.383	0.668

Abbreviations: CI, confidence interval; HR, hazard ratio.

^∗^ *p* < 0.05.

### 3.2. Gene Mutations in TCGA Data

Mutation set enrichment analysis was used to identify SNPs related to VEGF expression in the TCGA data. As shown in the waterfall plot in Figure 2a, *APC*, *TP53*, *KRAS*, *TP63*, *PIK3CA*, *FGF3*, *FGF12*, *FAT1*, *FGF19*, and *DNAH5* were the Top 10 genes whose mutations were associated with VEGF expression in the TCGA cohort. A Venn diagram (Figure 2b) was constructed to visualize the genes that were shared between the clinical NGS data and TCGA data; these genes included *TP53*, *TP63*, *FGF3*, *PIK3CA*, *FGF19*, *FAT1*, and *FGF12*.

Figure 2Potential gene signature shared by VEGF in the TCGA cohort and clinical patient population. (a) Waterfall plot of the Top 10 mutated genes in the ESCC TCGA cohort that express VEGF. (b) Venn diagram of the TCGA cohort and clinical patients.

**Figure:**
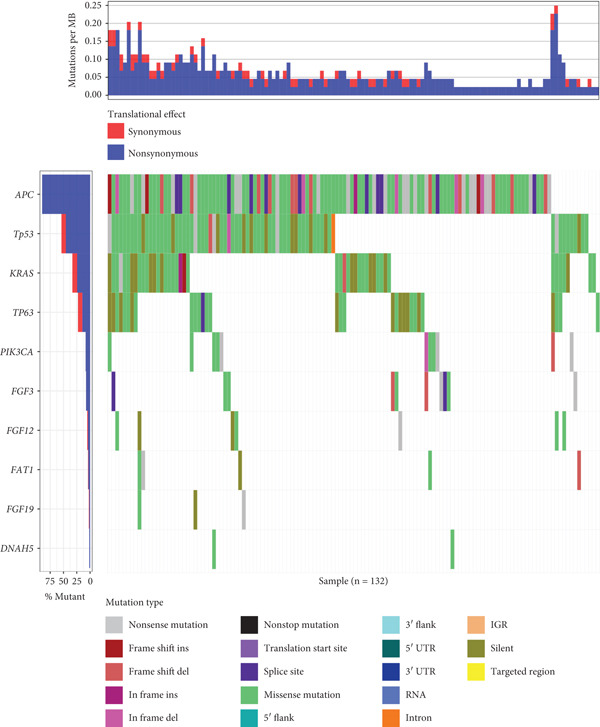
(a)

**Figure:**
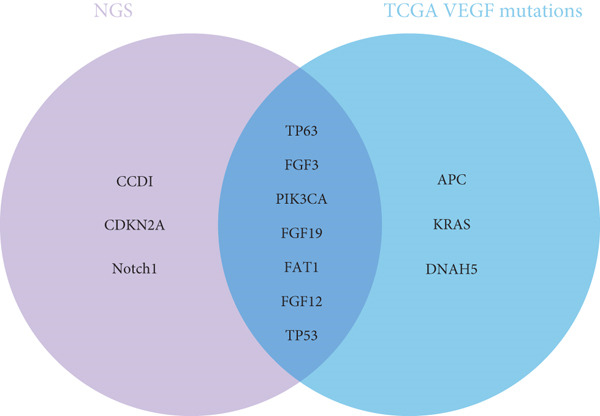
(b)

### 3.3. Survival Analysis Using the Gene Signature

K–M analysis and log‐rank tests were used to evaluate the prognostic value of the gene signature in the clinical cohort. Participants with mutations in signature genes had significantly lower RFS and OS than those without the signature (*p* = 0.0185 and *p* = 0.0232, respectively; Figure 3a,b). However, only *FAT1*, *FGF3*, *FGF12*, and *FGF19* mutations were significantly correlated with RFS (Figures 3c, 3d, 3e, 3f, 3g, 3h, and 3i). Cox analysis identified this four‐gene signature, clinical stage, M stage, and neutrophil count as independent prognostic factors for RFS (Table 2). Similar results were obtained via the use of PFS data from the TCGA cohort (Figure S1). A significant difference in prognosis was also observed between patients with and without VEGF overexpression.

Figure 3Survival analysis of the gene signatures. (a) RFS and (b) OS times of ESCC patients with or without the gene signature (*p* = 0.018 and 0.035, respectively). The RFS and OS time curves for (c) subjects with or without TP53 (with this mutation number (*n* = 46), *p* = 0.640); (d) TP63 (with this mutation number (*n* = 16), *p* = 0.421); (e) FGF3 (with this mutation number (*n* = 17), *p* = 0.041); (f) PIK3CA (with this mutation number (*n* = 10), *p* = 0.067); (g) FGF19 (with this mutation number (*n* = 14), *p* = 0.007); (h) FAT1 (with this mutation number (*n* = 14), *p* = 0.022); and (i) FGF12 (with this mutation number (*n* = 13), *p* = 0.038).

**Figure:**
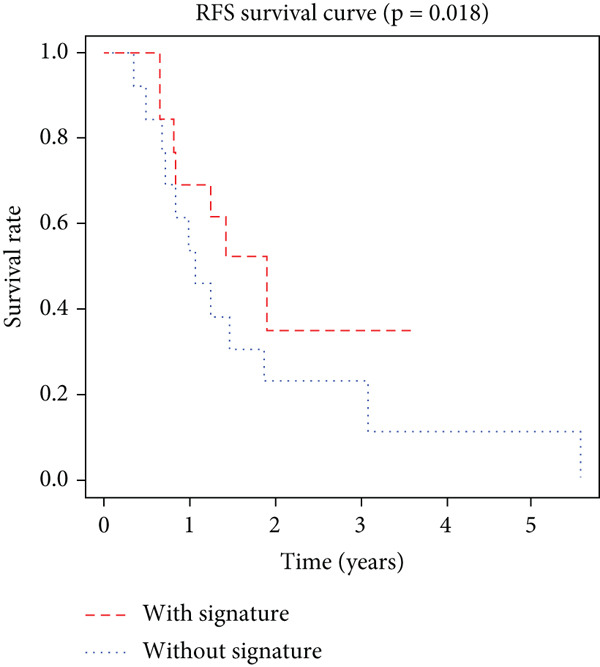
(a)

**Figure:**
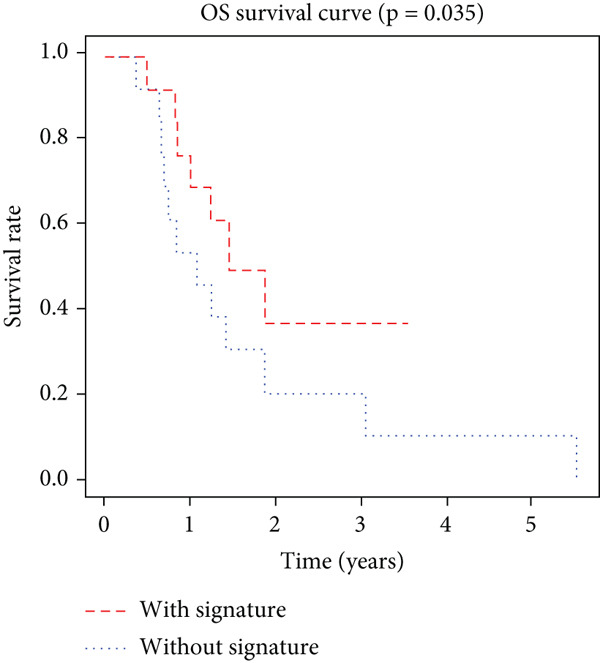
(b)

**Figure:**
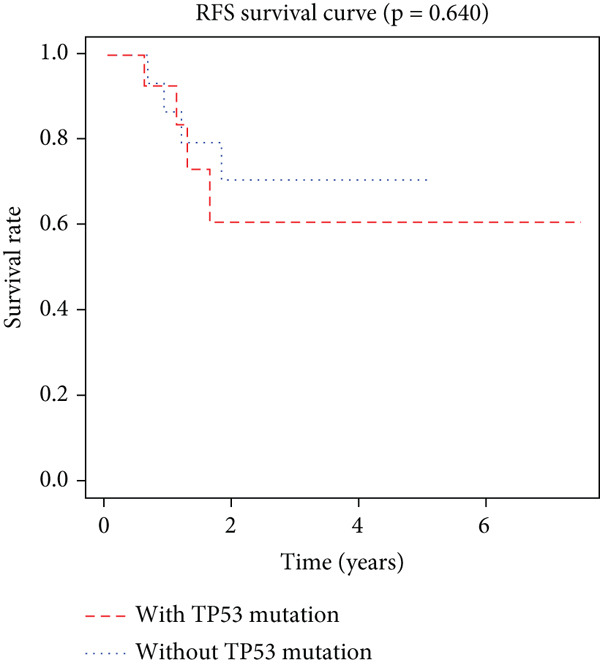
(c)

**Figure:**
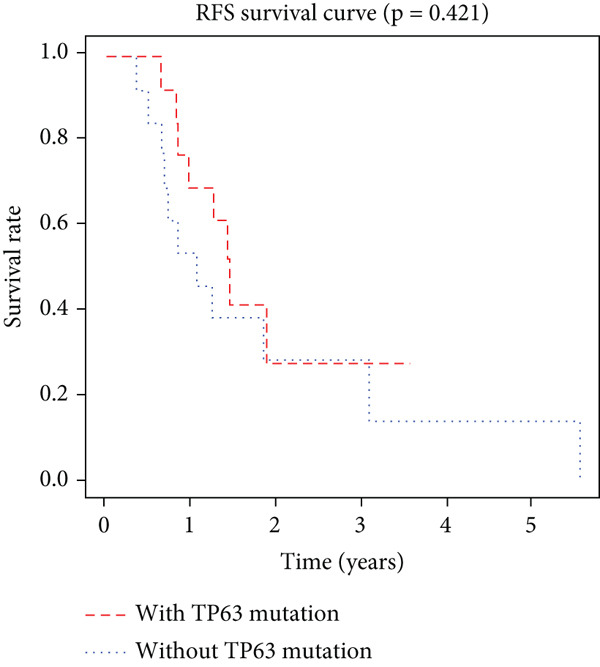
(d)

**Figure:**
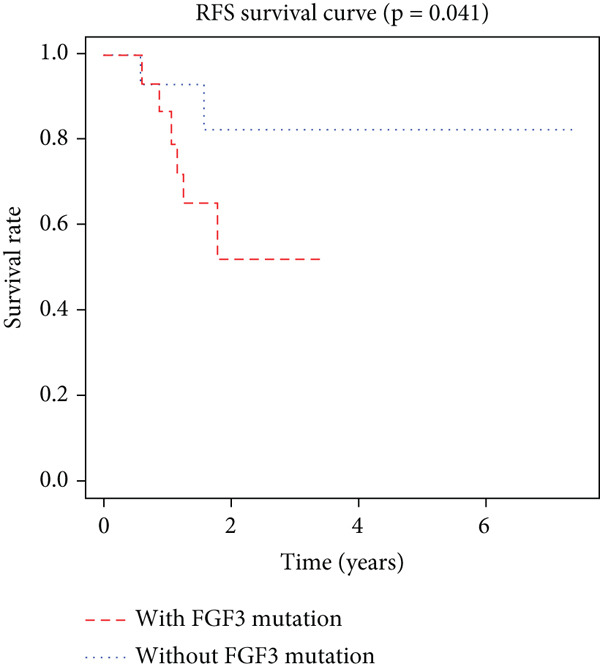
(e)

**Figure:**
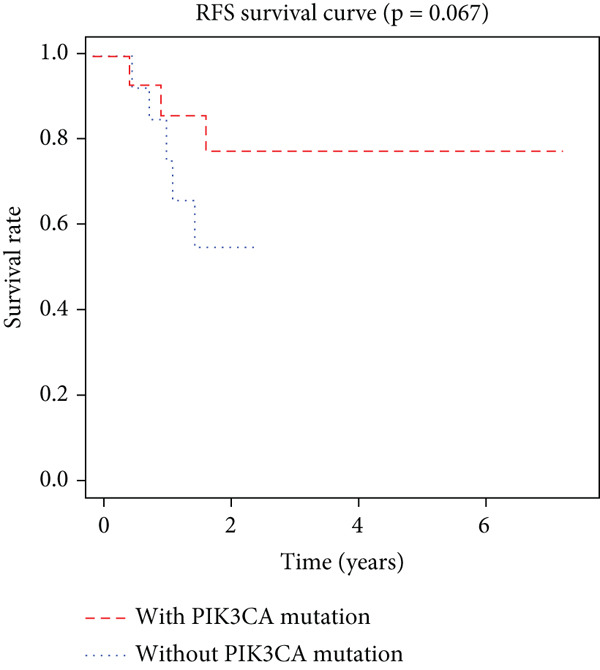
(f)

**Figure:**
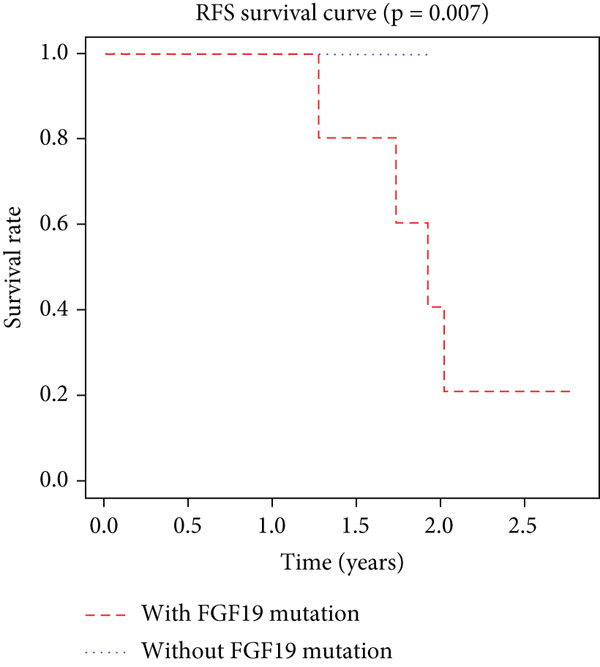
(g)

**Figure:**
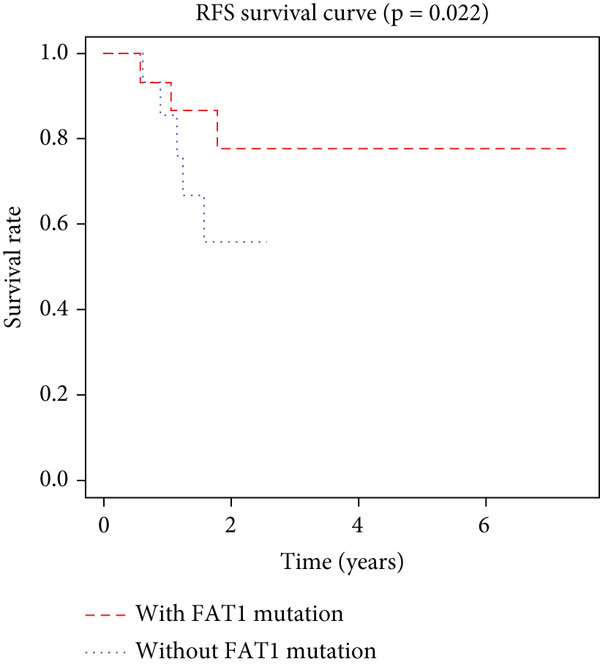
(h)

**Figure:**
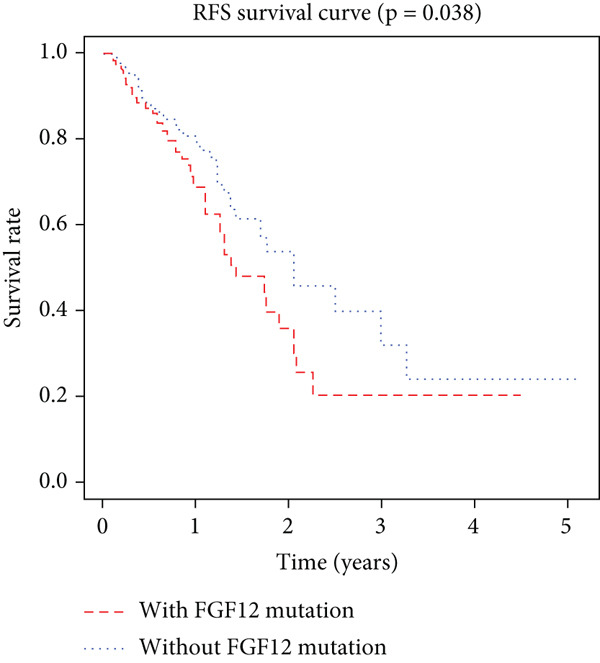
(i)

**Table 2:** Clinical–pathological characteristics in enrolled ESCC patients and their association with the gene signature.

**Variable**	**HR**	**Cl (95%)**	**p**
Univariate analysis (*n* = 50)			
Age			
< 60	1.000		
> 60	1.532	0.725–4.152	0.074
Gender			
Male	1.000		
Female	0.693	0.057–2.327	0.129
T stage			
Tis,T1–2	1.000		
T3–4	1.669	0.595–3.867	0.215
N stage			
N0	1.000		
N1–3	1.343	1.068–3.601	0.053
M stage			
M0	1.000		
M1	1.215	0.232–2.979	0.013 ^∗^
Clinical stage			
I–II	1.000		
III–IV	2.588	1.012–3.786	< 0.001 ^∗^
Neutrophil count			
1.5 × 10^9^ ≤ *n* < 2 × 10^9^	1.000		
≥2 × 10^9^	0.665	0.021–2.568	0.022 ^∗^
Gene signature mutation			
without	1.000		
with	1.212	0.077–2.336	0.042 ^∗^
Multivariate analysis (*n* = 50)			
Age			
<60	1.000		
>60	1.332	0.878–3.095	0.112
Gender			
male	1.000		
female	0.778	0.115–1.873	0.077
T stage			
Tis,T1–2	1.000		
T3–4	1.474	0.669–2.332	0.193
N stage			
N0	1.000		
N1–3	1.465	0.978–2.642	0.102
M stage			
M0	1.000		
M1	1.445	0.886–1.789	0.029 ^∗^
Clinical stage			
I–II	1.000		
III–IV	2.337	1.258–4.078	< 0.001 ^∗^
Neutrophil count			
1.5 × 10^9^ ≤ *n* < 2 × 10^9^	1.000		
≥2 × 109	1.012	0.752–2.666	0.038 ^∗^
Gene signature mutation			
Without	1.000		
With	1.372	0.717–2.834	0.029 ^∗^

Abbreviations: CI, confidence interval; HR, hazard ratio.

^∗^ *p* < 0.05.

### 3.4. SVM‐RFE Analysis and Construction of a Predictive Model

The application of the SVM‐RFE algorithm revealed that *FAT1*, *FGF3*, *FGF12*, and *FGF19* expressions; neutrophil count; and M stage were most significantly associated with prognosis in ESCC patients (Figure 4a). Logistic regression was then used to evaluate the prognostic value of the gene signature and various clinical characteristics (Figure 4b). *FAT1* mutation was negatively correlated with ESCC patient prognosis; however, the other factors were positively correlated (*p* < 0.05). ROC curves for the model were generated, and area under the curve (AUC) values for 5‐year OS were compared between participants with and without the identified risk factors (*FAT1*, *FGF3*, *FGF12*, and *FGF1*9 mutation; neutrophil count; and M stage); the AUC and *C* values were 0.884 and 0.7662, respectively (Figure 4c,d). Finally, we constructed a nomogram to report the effects of the six variables and VEGF expression on the prognosis of patients with ESCC (Figure 4e). These findings indicated that the model could effectively predict ESCC patient outcomes.

Figure 4Construction of the predictive model. (a) SVM‐RFE selection of the gene signature and clinical characteristics. (b) Hazard ratios and *p* values of the patients included in the multivariate logistic regression model. (c) The ROC curve of the predictive model. (d) Calibration curves of the model. (e) The nomogram indicates the risk score of the selected variables and *Vegf* expression.

**Figure:**
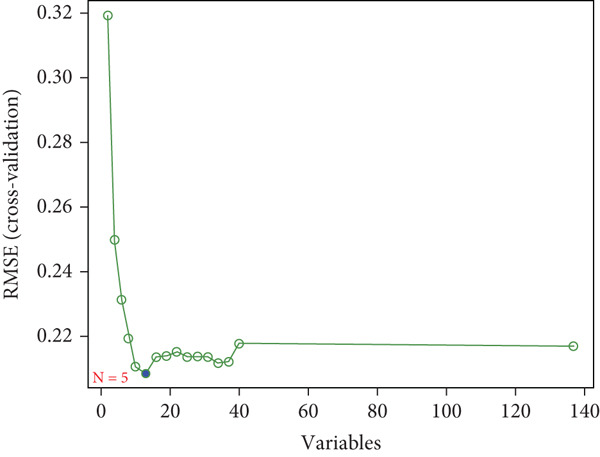
(a)

**Figure:**
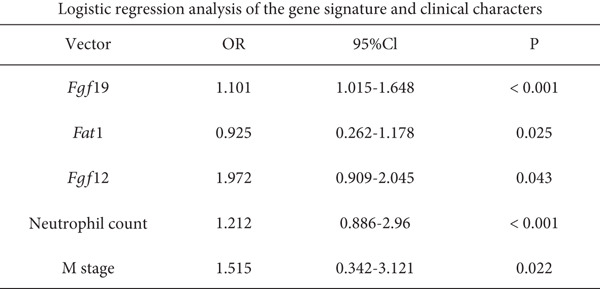
(b)

**Figure:**
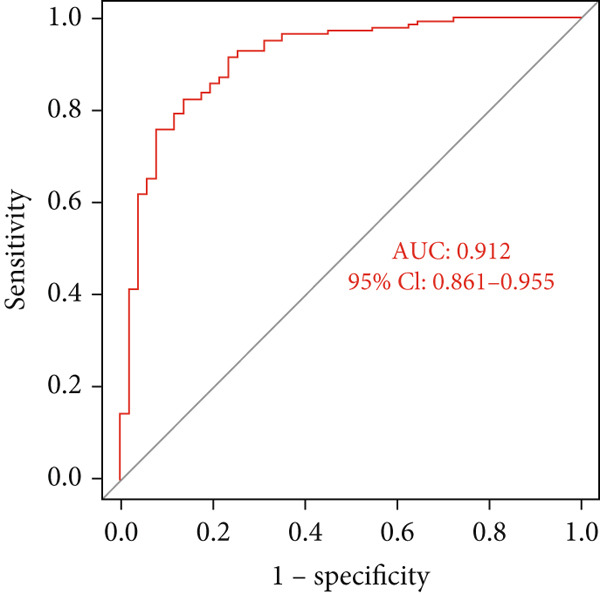
(c)

**Figure:**
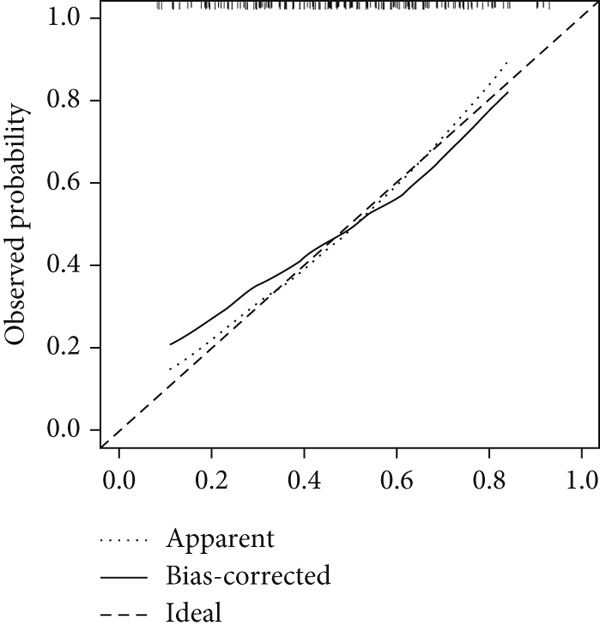
(d)

**Figure:**
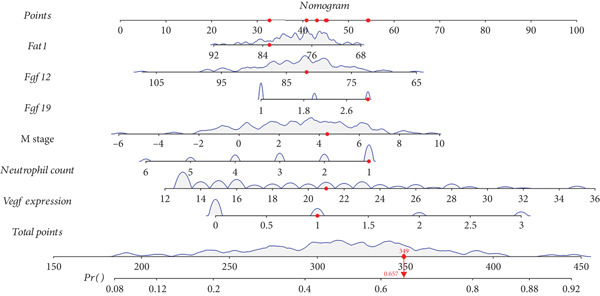
(e)

### 3.5. GSEA

To explore potential mechanisms associated with the gene signature, we used an online GSEA tool to identify enriched KEGG pathways in the TCGA expression data. A total of 323 KEGG pathways were closely associated with the gene signature, of which the Top 5 were related to VEGF: mitogen‐activated protein kinase, regulation of the actin cytoskeleton, transforming growth factor‐beta, and extracellular matrix–receptor interaction (Figures 5a, 5b, 5c, 5d, and 5e). These findings indicate that signature genes are particularly associated with pathways related to cancer‐associated fibroblasts (CAFs) and reshaping of the extracellular microenvironment.

Figure 5GSEA results of the expression profile of patients with the gene signature. The Top 5 enriched terms are associated with the (a) VEGF‐related, (b) MAPK, (c) regulation of the actin cytoskeleton, (d) TGF‐beta, and (e) ECM receptor interaction pathways.

**Figure:**
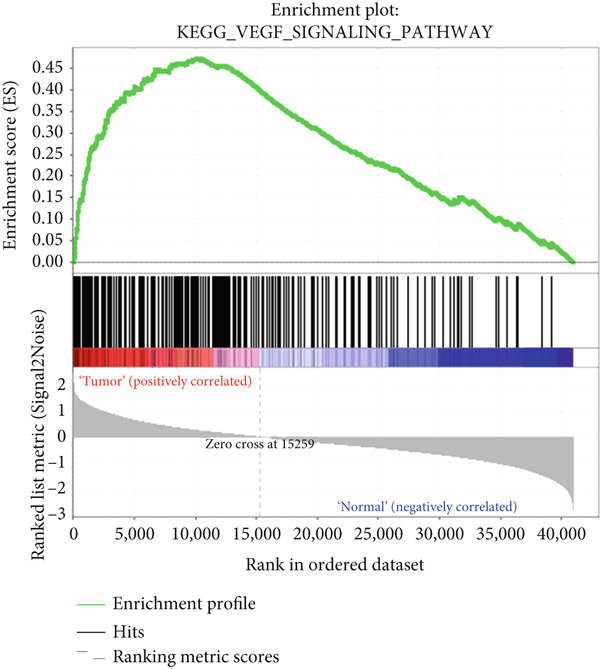
(a)

**Figure:**
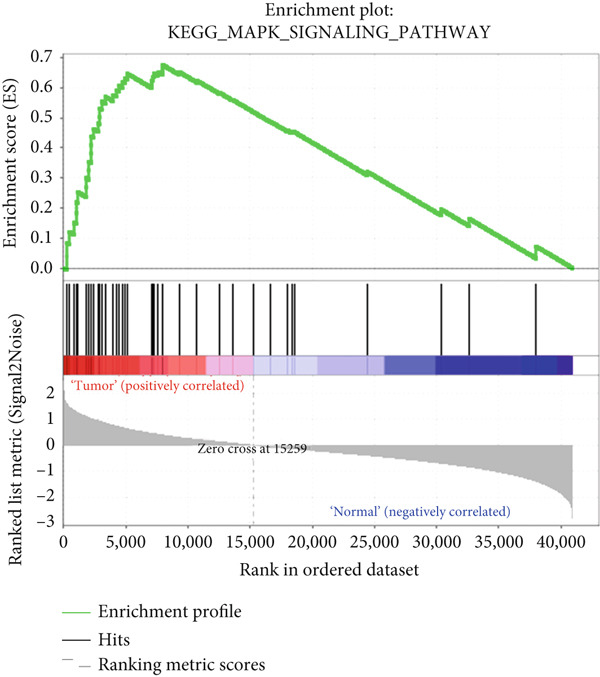
(b)

**Figure:**
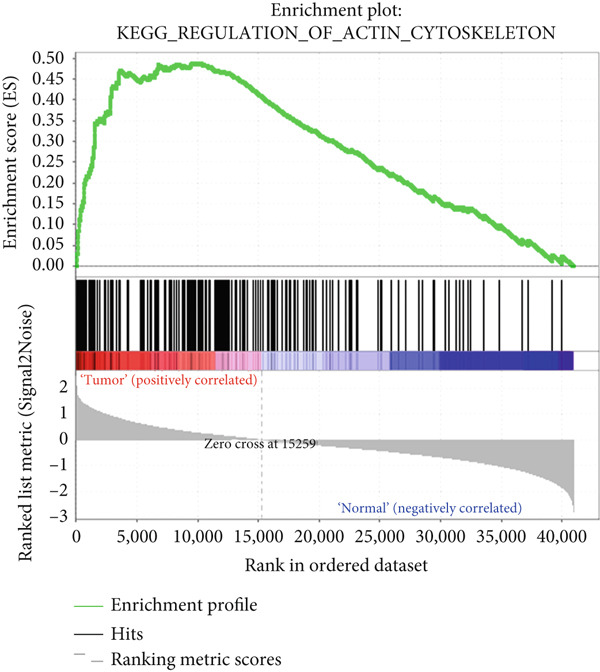
(c)

**Figure:**
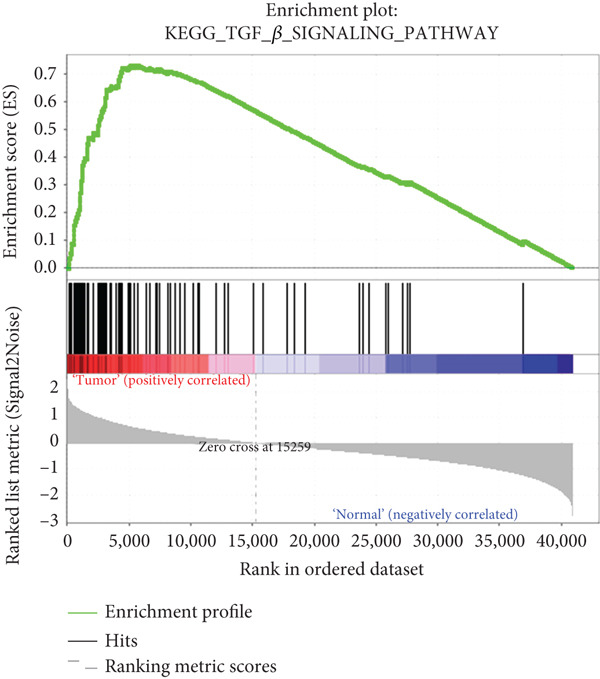
(d)

**Figure:**
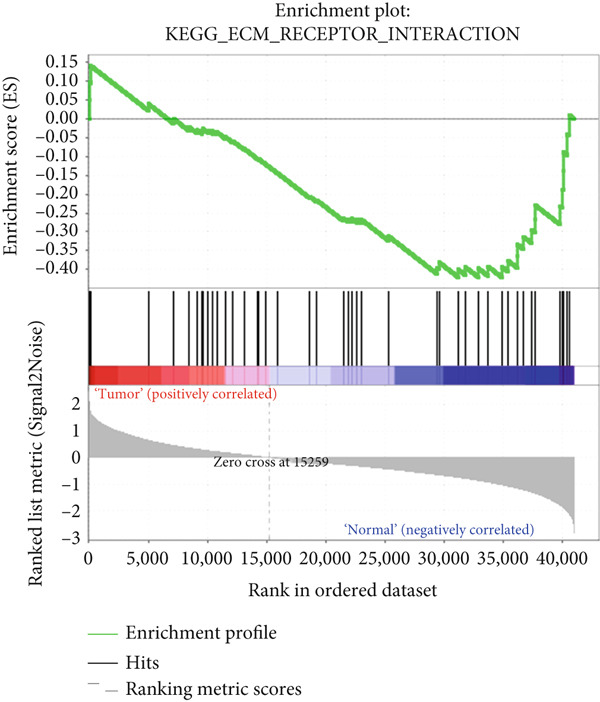
(e)

## 4. Discussion

In recent decades, several new treatments have been developed for ESCC; however, the survival rates and clinical outcomes of this patient population remain poor [3]. A characteristic of advanced ESCC is the invasion of new blood vessels [15]. Thus, the overexpression of VEGF, a biomarker of angiogenesis, is considered to be predictive of more aggressive disease [16]. However, although effective drugs that target the VEGF pathway have been developed for several cancers, including lung, breast, liver, and colorectal cancer, the specific role of VEGF in ESCC has remained unclear [17]. Thus, further elucidation of the function of VEGF and its underlying molecular mechanisms in ESCC is needed.

In the present study, we assessed the presence of gene mutations in clinical patients and a TCGA cohort with VEGF expression and identified a signature of genes that were enriched primarily in CAFs and microenvironment‐related pathways. CAF‐induced reshaping of the microenvironment has been shown to be the major factor associated with ESCC progression in the context of VEGF expression. The transformation of fibroblasts into CAFs plays a major role in the creation of an immunosuppressive microenvironment for cancer cells [17–19], and several studies have shown that the abundance of CAFs is closely correlated with the prognosis of ESCC patients [20, 21]. However, although the FGF–FGF receptor (FGFR) signaling cascade is an important driver of cancer growth, only a subset of patients benefit from treatment with agents that block FGF–FGFR signaling [22]. Similar variations in clinical response have been observed in patients receiving therapies targeting the epidermal growth factor receptor, human epidermal growth factor receptor 2, or VEGF [23]. These findings suggest that cross‐talk between these signalling pathways may constitute a bypass circuit that increases cancer cell–specific drug resistance. Thus, the development of effective target drugs requires the identification of hub genes associated with various pathways. The present study characterized a set of new hub genes (*FAT1*, *FGF3*, *FGF12*, and *FGF19*) associated with the FGF and VEGF pathways. Notably, *FGF12*, which was among the hub genes identified here, was recently identified as a novel biomarker for ESCC prognosis [24].

VEGF expression is correlated with vessel density and other specific clinical characteristics. Therefore, the prognostic value of the gene signature identified here may depend on characteristics such as the specific tumor stage or clinical status of the patient. To quantify this, we constructed a predictive model by combining the gene signature with other clinical characteristics of ESCC patients. A machine learning diagram, the SVM‐FEM, was used to identify a four‐gene signature that could help to predict individual OS and guide the clinical management of ESCC patients. Our model also identified the neutrophil count as an independent prognostic factor in ESCC. An elevated neutrophil‐to‐lymphocyte ratio is considered indicative of an immunosuppressive microenvironment in cancers [25]. The neutrophil count is also reportedly associated with VEGF expression in digestive system cancers [26]. In addition, increased expression of FGFs contributes to neutrophil chemotaxis and favors the transvascular migration of neutrophils [27]. Therefore, the neutrophil count is expected to vary dynamically with the other variables in the gene signature.

GSEA revealed enrichment of CAF‐related pathways in the TCGA cohort. There is a complex cross‐talk mechanism between CAFs and cancer cells [28], and several important secreted factors, including FGFs, can mediate tumorigenesis. Cancer cells also reshape CAFs via a feedback mechanism [29], and various CAF subpopulations may have either tumor‐promoting or tumor‐suppressive activities [30]. The novel gene signature identified in this study could be used to classify CAFs or other extracellular matrix cells, in addition to its prognostic role. However, more laboratory and clinical work is needed to validate the various applications of our signature.

## 5. Conclusions

The model generated and validated in this study could be used to assess individual patients′ risk on the basis of their scores for each model factor. Further research using a larger patient cohort is needed to determine whether these risk scores are associated with different clinical responses or outcomes.

## Ethics Statement

The present study was approved by the Ethics Committee of the Affiliated Hospital of Hebei University. The research was performed in accordance with the World Medical Association Declaration of Helsinki.

## Disclosure

All authors agreed to submit the final manuscript to the current journal. Haibo Wang and Tingting Li agree to be accountable for all aspects of the work.

## Conflicts of Interest

The authors declare no conflicts of interest.

## Author Contributions

All the authors made substantial contributions to the study conception and design and the acquisition, analysis, and interpretation of the data. Yonghui Li and Ruiyao Wang drafted the article and revised it critically for important intellectual content. Haibo Wang and Tingting Li conducted the statistical analysis. Cheng Long Zhang and Shaoyong Dong interpreted the data. Cheng Long Zhang and Biao Zhang checked the data in the manuscript. Haibo Wang revised the final manuscript. Yonghui Li and Ruiyao Wang are equal contributors.

## Funding

This study was supported by the Baoding Science and Technology plan project, 2241ZF331.

## General Statement

*Conclusion*. This study constructed a four‐gene signature‐based prognostic model for ESCC. The model effectively stratified ESCC patients with multiple risk factors.

## Supporting information


**Supporting Information** Additional supporting information can be found online in the Supporting Information section. Figure S1: Progress‐free survival analysis of the gene signature in the TCGA cohort. PFS times of 133 TCGA‐originated ESCC patients with or without gene signatures. (a) The number of subjects with/without FAT1 mutations (15, *p* = 0.035). (b) The number of subjects with/without FGF19 mutations (27, *p* = 0.041). (c) The number of subjects with/without FGF12 mutations (29, *p* = 0.044). (d) The number of subjects with/without FGF3 mutations (66, *p* = 0.038).

## Data Availability

The datasets used and/or analyzed during the current study are available from the corresponding author upon reasonable request.
