# Supplementary figures and images for: New Gene Signature–Based Prognostic Model for Patients With VEGF‐Overexpressing Esophageal Squamous Cell Carcinoma
Source: Biomed Res Int. 2025 Dec 7;2025:5694628. doi: 10.1155/bmri/5694628 (PMC12682582; doi:10.1155/bmri/5694628)

**A**FAT1 ( $p=0.035$ )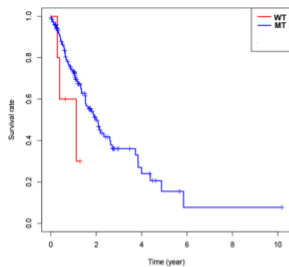**B**FGF19 ( $p=0.041$ )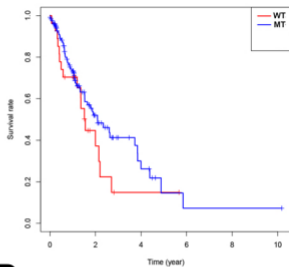**C**FGF12 ( $p=0.044$ )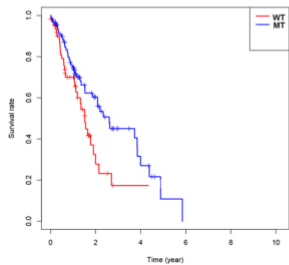**D**FGF3 ( $p=0.038$ )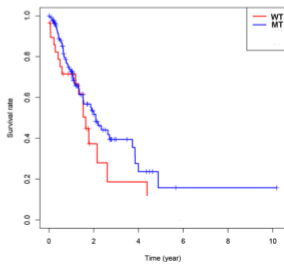

Supplement: Supplementary file 1 — Supporting Information Additional supporting information can be found online in the Supporting Information section. Figure S1: Progress‐free survival analysis of the gene signature in the TCGA cohort. PFS times of 133 TCGA‐originated ESCC patients with or without gene signatures. (a) The number of subjects with/without FAT1 mutations (15, p = 0.035). (b) The number of subjects with/without FGF19 mutations (27, p = 0.041). (c) The number of subjects with/without FGF12 mutations (29, p = 0.044). (d) The number of subjects with/without FGF3 mutations (66, p = 0.038). [file BMRI-2025-5694628-s001.pdf]
